# Introduction of bundle of care and effect on surgical site infections in patients taken for elective surgical procedures

**DOI:** 10.1186/cc14034

**Published:** 2014-12-03

**Authors:** A Waheed, S Bhat, M Parvez

**Affiliations:** 1 Department of Anesthesia, District Hospital Doda (Deemed Medical College), Jammu and Kashmir, India; 2 Department of Gynecology, District Hospital Doda (Deemed Medical College), Jammu and Kashmir, India; 3 Department of Surgery, District Hospital Doda (Deemed Medical College), Jammu and Kashmir, India

## Introduction

Surgical site infection (SSI) previously termed postoperative wound infection is defined as that infection presenting up to 30 days after a surgical procedure if no prosthetic is placed and up to 1 year if a prosthetic is implanted in the patient. Several recommendations have been published (skin preparation, surgical antibiotic prophylaxis, control of the OR environment and improvements in the surgical technique) to improve the patient safety and quality of care in the OR. Use of the bundle of care to improve patient outcomes is becoming more widespread; however, their use is more common internationally than in India.

## Methods

A surveillance study for SSI after routine surgical procedures was conducted from September 2012 until August 2014. A bundle of care consisting of five elements covering the surgical process was introduced in September 2013. The elements of the bundle were perioperative antibiotic prophylaxis, hair removal before surgery, perioperative normothermia, perioperative euglycemia and operating room discipline. Normothermia was defined as a temperature between 36.0 and 38.0°C. Euglycemia was defined as blood glucose <180 mg/dl. Antibiotic prophylaxis was given 15 minutes before the incision. Hair removal whenever needed was done with clippers. Theatre discipline was counted for following points: permanent wearing of scrub suits, surgical cap and mask covering the nose and mouth by all persons in the OR during the surgical procedure. The incidence of SSI was studied as a primary outcome. Morbidity/mortality for next 3 months was studied as the secondary outcome.

## Results

Implementing the bundle of care led to a decline in infection rate from 15% before the intervention to 11.4% after the introduction of bundle of care, a fall of 27%, which is significant (*P *< 0.001). No significant difference in 3-month mortality was found. The compliance to this bundle of care also steadily increased from 10% in September 2013 to 55% in August 2014. For 542 surgical procedures during the study period, 62 SSI (11.4%) occurred as compared to 102 cases for 680 in the control period (15%). The adjusted odds ratio of the SSI rate was 0.7319 and was found to be 27% lower post intervention.

## Conclusion

The implementation of the bundle was associated with improved compliance over time and a significant reduction of the SSI rate. This makes the bundle an important tool to improve patient safety.

